# The Effects of Anthropomorphism, Message Framing, and Voice Type on Unhealthy Sleep Behavior in Young Users: The Mediating Role of Risk Perception

**DOI:** 10.3390/ijerph19159570

**Published:** 2022-08-04

**Authors:** Ying Li, Yanfei Zhu, Guanqun Zhang, Junliang Zhou, Jinlai Liu, Zhuoxin Li, Boqian He

**Affiliations:** 1 Department of Art and Design, Beijing University of Chemical Technology, Beijing 102206, China; 2 School of Mechanical Engineering, Southeast University, Nanjing 211189, China

**Keywords:** sleep health, anthropomorphism design, message framing, voice type, interaction

## Abstract

Insufficient sleep is a severe social public health problem that can adversely affect the physical and mental health of young people. This study examined risk perceptions for unhealthy sleep behaviors and intentions for healthy sleep behaviors under different combinations of anthropomorphism, message framing, and voice type in cartoons. We used a three-factor between-subject experiment of two (anthropomorphism: anthropomorphic vs. non-anthropomorphic) × two (message framing: positive frame vs. negative frame) × two (voice type: cartoon child voice vs. adult female voice) design. We examined the effects of different audiovisual combinations of cartoon attitude, risk perception, and behavioral intention and the mediating role of risk perception. The research results show that (1) the integration of anthropomorphic design elements can positively impact users’ attitudes toward cartoons; (2) when the interface information is presented in a negative frame, anthropomorphism can more positively influence users’ attitudes toward cartoons than non-anthropomorphism; and (3) anthropomorphism, message framing, and voice type in cartoons significantly interact with risk perception. In addition, risk perception mediates the influence of anthropomorphism, message framing, and voice type on behavioral intention.

## 1. Introduction

Insufficient sleep is becoming a serious health risk [1,2]. It causes weight gain [3] and obesity [4], cardiovascular and cerebrovascular diseases, coronary heart disease [5], mood disorders [6], and depression [7,8]. As an essential part of the life cycle, sleep is an important bodily function vital to human health [9,10]. Healthy sleep habits benefit our brain, mental health, and cardiovascular and immune systems [10].

To draw attention to the importance and quality of sleep, the International Foundation for Mental Health and Neuroscience launched a global campaign called World Sleep Day in 2001 [11]. The Sleep Research Society recommends that adults aged 18–60 regularly get at least 7 h of sleep per night [12]. There is much evidence that sleep deprivation can severely affect young adults’ physical and mental health [6,13,14]. However, young adults are also at high risk for unhealthy sleep [15,16,17]. Physiologically, sleep-deprived young adults are more likely to develop chronic diseases such as diabetes, hypertension, and myocardial infarction [13,18]. Furthermore, sleep-deprived young adults are also more likely to experience psychological effects such as mood disturbances [15,19], increased stress [20], symptoms of depression [21], and even suicidal thoughts [22]. Over time, chronic sleep deprivation can lead to excessive daytime sleepiness, inattention, emotional regulation disorders, and increased risk-taking behavior in young adults [15,23,24,25,26].

In investigating the reasons for insufficient sleep, we found that unreasonable time allocation and increased interactive entertainment products lead to insufficient sleep or procrastination in young people [14,27,28,29]. Specifically, the richness of entertainment product interfaces often causes users to delay or shorten their normal sleep time due to content addiction. This has direct consequences such as disordered physiological cycles and increased negative emotions the next day; over time, this forms a vicious cycle [15,30]. Therefore, how to effectively prevent addiction is an important question, and how to organically restrain entertainment information persuasion into interactive products has become an urgent issue at the intersection of design and public health.

Behavioral interventions are a common approach to improving unhealthy sleep among young adults [6,31,32]. Experts note that the transition from adolescence to adulthood may be the best time to intervene in negative health behaviors to prevent future risk of disease [16,33]. In addition, according to the health belief model, unhealthy sleep behavior can be effectively intervened by increasing young adults’ perceptions of the physical and mental risks and severity of unhealthy sleep [34,35]. Anthropomorphism is a more vivid way to describe the risks [36]. It is thought to have the potential to enhance a viewer’s risk perception of disease [34], thereby intervening in unhealthy behaviors. Anthropomorphism has emerged as a novel, exciting, effective, informational intervention. For example, anthropomorphism has been used to convey health risks in the context of COVID-19 [37], PM2.5 [34], drugs [38], harmful light [34], enterovirus [34], health website information [39], and insects [34]. However, we found that research on anthropomorphism in sleep is still scarce. Previous studies on health persuasion message design identified risk perception and behavioral intentions as essential factors in implementing/influencing protective behaviors [40,41]. Steinmetz et al. [42] and Branscum et al. [43] also suggested that healthy sleep behavior interventions should be informative (e.g., advantages/disadvantages of getting healthy sleep), persuasive, and socially encouraging.

Therefore, this study explores effective combinations of interactive persuasion information design through factorial experiments. specifically, we carried out a three-factor experiment: (anthropomorphism: anthropomorphic vs. non-anthropomorphic) × (message framing: positive frame vs. negative frame) × (voice type: cartoon child voice vs. adult female voice). The dependent variables were subjectively measured by three Likert scales: animation attitude, risk perception, and behavioral intention. The purpose was to investigate the effects of different combinations of anthropomorphism, message framing, and voice type on cartoon attitudes, risk perception and behavioral intention.

## 2. Literature Review

### 2.1. Anthropomorphism Design

The essence of anthropomorphism is the imaginary behavior of nonhuman beings such as gods, animals, or objects endowed with human characteristics, motivations, intentions, and emotions [44]. It is often used to rationalize the behavior of animals or inanimate objects [45]. Anthropomorphism adds human features to nonhuman entities to amplify human personification tendencies [34]; this includes adding physical characteristics (e.g., eyes, mouth, and expressions [46,47]), adding image features (e.g., doctor images to interactive health websites [39]), and designing two points and a parting line for intelligent speakers [48].

Anthropomorphism can spontaneously develop vital personal relevancy and emotional responses to inanimate objects [49,50]. Specifically, the anthropomorphism effect can be further enhanced when the audience perceives the information as relevant to itself, producing an emotional response [36], resulting in favorable outcomes. Muzumdar et al. [38] and Block and Keller [51] found that viewing an anthropomorphic drug image positively affected attitudes toward a particular message. Burgoon et al. [52] found that participants showed increased interest in a task interface with more positive attitudes when more anthropomorphic faces were present in the interface. Romero and Lado [37] found that using anthropomorphic robots to prevent COVID-19 inspired better customer attitudes and higher hotel booking intentions. However, Huang [34] found that anthropomorphizing enteroviruses into an image with a crying face weakened the positive effect of anthropomorphism on behavioral intention. Based on the research basis of anthropomorphism in the above fields, we propose the following hypothesis:

**Hypothesis** **1** **(H1).** *Anthropomorphism of a healthy sleep persuasion interface can arouse a more positive cartoon attitude among young users (H1a) but cannot promote the viewer’s behavioral intention for healthy sleep (H1b).*

### 2.2. Message Framing

The framing effect refers to people’s different decision-making judgments caused by the description of an objectively identical problem [53]. The risk selection framework is called positive or negative because its information is often presented in positive versus negative descriptions. Regarding health problems, the positive frame highlights the potential benefits of performing healthy behaviors, while the negative frame highlights the negative consequences of not performing healthy behaviors [54]. As a common persuasion strategy, positive and negative framing is often used to promote desirable healthy behaviors [55,56,57] and communications.

In researching message framing in healthy behavior persuasions, we found that although some studies have shown that positive (gain)/negative (loss) frames can generate better attitudes or higher behavioral intentions [55,58], more evidence suggests an overall small or negligible effect [59,60,61,62]. For example, de Bruijn et al. [63] conducted a study on the effect of message framing on adolescents’ hearing loss prevention intentions and found no apparent main effect on behavioral intentions. In their study, Goh et al. [56] did not observe significant differences in message framing regarding Type 2 diabetes (T2D) risk perceptions or behavioral intentions. Arora et al. [64] found that message framing had no significant effect on attitudes and intentions to exercise and fitness. This suggests that the effectiveness of gain versus loss may be mediated by other variables [57].

Given that many studies have shown that anthropomorphic faces and voices in an interface can stimulate positive attitudes in participants [52,65], we suspect that anthropomorphism can also stimulate the influence of message framing on cartoon attitudes.

Regarding behavioral intentions, only one study examined the interaction of anthropomorphism and information framing [47]. Karpinska-Krakowiak et al. [47] found that anthropomorphism and message framing interact in promoting green intentions. Combined with the findings from Huang’s [34] promotion study on health products, if the victim is given a crying face, the positive effect of anthropomorphism on behavioral intentions is attenuated due to less activated disease threat perception. However, our research did not find studies on the interaction of anthropomorphism and message framing in the health domain. Nevertheless, based on the above literature, we put forward the hypothesis:

**Hypothesis** **2** **(H2).** *There is no significant relationship between cartoon attitude and behavior intention when viewing positive or negative frame information.*

**Hypothesis** **3** **(H3).** *Message framing and anthropomorphism have a significant interaction with cartoon attitudes (H3a) but no significant interaction with healthy sleep behavior intention (H3b).*

### 2.3. Voice Type

Voice is the friendliest and most natural way to communicate with people and machines [66,67]. As the primary communication tool to convey information, voice can influence people’s attitudes toward advertising [68], purchase intention [69], and purchase behavior [70]. In health interventions, however, we did not find a role for voice intervention alone. However, multimedia presentations that combine audio and visual design factors are thought to provide persuasive behavioral health interventions [71].

Among them, there are two ways to transmit health information through voice [72]: One is to combine the voice channel with the message framing, that is, to present the content of the voice in a positive/negative manner. For example, Schneider et al. [71] explored the effects of visual and auditory message framing on smoking. Their study presented subjects with visual images and audio-dubbed videos of smoking that were programmed to be gains or losses. The study found that using a gain framework (i.e., advocacy of smoking cessation) should be considered more when promoting preventive behaviors to avoid smoking (i.e., the benefits of not smoking). The other voice method is through sound channel parameter information such as timbre and pitch [73,74]. Previous research has shown that when we speak, we use various vocal and visual cues to communicate our thoughts and emotions [75]. Children’s voices, with fundamental frequencies ranging from 200 to 320 Hz, are associated with innocence, extroversion, and curiosity [72]. The fundamental frequency of adult female voices is between 120–480 Hz and is often associated with affinity and empathy [72]. However, whether different types of sounds and visual cues provided by anthropomorphic nonhuman entities can also convey the thoughts and emotions we want to express when we watch interactive cartoons has not been studied. Therefore, we used two voice types (cartoon children and adult female) to establish matching relationships with anthropomorphic/non-anthropomorphic visual cues and positive/negative text and voice content to study the effects of anthropomorphism, message framing, and voice type on the effects of cartoon attitudes, risk perceptions, and behavioral intentions. Specifically, we hypothesized:

**Hypothesis** **4** **(H4).** *Viewers exposed to cartoon children voices will have better attitudes (H4a) and higher risk perception (H4b) toward anthropomorphism (compared with non-anthropomorphism) when the message framing is positive rather than negative and will show more positive behavioral intentions (H4c).*

### 2.4. The Mediating Role of Risk Perception

Risk perception, as a highly correlated concept, can act as a mediator [55,76,77], and risk perception variables are important in promoting behavioral intentions [76]. As Huang [34] showed, the greater the perceived harm of nociceptive infection, the stronger the perceived threat of disease (i.e., the mediator) and the stronger the protective behavioral intention. In addition, message framing is a significant predictor of risk perception [78]. Risk perception can mediate the effectiveness of message framing and other variables on behavioral intention [58]. For example, Jin and Atkinson [79] proved that risk perception has a positive mediating effect on the influence of emotion and message framing on behavioral intentions, and Gursoy et al. [58] proposed a message framing and information appeal in changing perceived vaccines risk.

This study proposes that the perceived risk of unhealthy sleep may be an important underlying mechanism of anthropomorphism, especially when matched to message framing and voice type. Therefore, we hypothesize:

**Hypothesis** **5** **(H5).** *Risk perception of unhealthy sleep mediates the interaction effects of message framing, voice type, and anthropomorphism on behavioral intentions.*

## 3. Methodology

This study used a three-factor between-subject experiment of 2 (anthropomorphism: anthropomorphic vs. non-anthropomorphic) × 2 (message framing: positive frame vs. negative frame) × 2 (voice type: cartoon child voice vs. adult female voice) design. The three dependent variables of cartoon attitude, risk perception, and behavioral intention were subjectively measured on seven-point Likert scales. The main effects and interaction effects among the three independent variables of anthropomorphism, message framing, and voice type were examined. We used Hayes [80] PROCESS SPSS macro to test whether risk perception mediates the interaction effects of message framing, voice type, and anthropomorphism on behavioral intentions.

### 3.1. Participants

To ensure that the experimental materials and the quality of the questionnaire data were fully understood, our questionnaires was mainly distributed to users with a bachelor’s degree or above. In this study, a total of 222 Chinese students were recruited to participate in our online experiment through the Questionnaire Star platform (116 males and 106 females; 208 with a bachelor’s degree or above). All participants received a small gift as a reward after completing the questionnaire.

### 3.2. Design

To ensure that our research was in line with the interaction situation to meet the external validity, we designed the experimental stimulus to resemble a pop-up cartoon in the interaction. We designed eight cartoons to explore the effects of anthropomorphic graphics, message framing language, and voice type. We used different combinations to study unhealthy sleep behavior (specifically insufficient sleep) among young users sleeping late (see Appendix A for static images; see Supplementary Materials for experimental stimulus).

The design of the persuasive cartoon included two parts, visual and auditory. The visual design was realized by manipulating anthropomorphic graphics and positive and negative frames. The Chinese character information and the auditory design were realized using voice type; see Table 1 for the different combinations of pictures and text parts. In the design of anthropomorphic graphics, facial features (eyes and expressions) can serve as sufficient cues to give inanimate objects human-like qualities [47]. We drew on previous studies that used facial features to induce anthropomorphic thinking [46,47,81]. Thus, in the anthropomorphic condition, we depicted the alarm clock and the moon as having eyes and expressions, and in the non-anthropomorphic state, without eyes and expressions. To further enhance the anthropomorphic look and feel, human-like features were added for the anthropomorphic conditions (e.g., “the alarm clock stretches its arms”, “the red bloodshot eyes of the alarm clock”, “sleep symbol: ZZZ”, the moon’s nightcap).

Regarding the design of Chinese character information for positive and negative frames, we showed positive outcomes of adopting healthy behaviors (good sleep is good for physical and mental health). Similarly, the structure of Chinese character information in cartoons involving negative frames was the same but informed about the negative consequences of unhealthy behavior (lack of sleep is harmful to physical and mental health). In summary, positive frames contain positive descriptions and positive outcomes, while negative frames contain negative words and outcomes.

For voice type, differences in rhythm and intonation of recorded sounds compared with synthesized speech may lead to experimental error [72]. Therefore, we adopted a text-to-speech system in video editing software to produce samples of both speech types. The current research uses Chinese, and the content of the phonetic text is similar to the above positive and negative framing of Chinese character information. The positive frame uses the text described in the positive (healthy sleep, developing a scientifically regular life, and rest, which is beneficial to physical and mental health). In contrast, the negative frame poses lack of sleep, inability to develop a scientifically regular life, and rest as harmful to physical and mental health. Voice types are mainly distinguished by timbre. The cartoon children’s voices use “anime Xiaoxin” audio, and the adult female voice uses “intellectual female voice” audio.

To guarantee satisfactory internal validity and reduce potential confusion [47], we designed the persuasion cartoons to be as simple as possible. Specifically, according to the above design scheme, we had four groups of cartoon scripts with similar structures. Each script included four static interfaces, and each group of static interfaces maintained image similarity. To achieve the matching and synchronization of visual effects and auditory senses [71], we used short video clips to match two voices with four sets of interfaces, producing eight collections of 12 s persuasive cartoons. We set the positive frame text to gradually lighten to encourage falling asleep slowly with the voice prompt. The negative frame text progressively deepened, which means staying up late without following the voice prompt. These design options and strategies allowed us to maintain internal and external validity and eliminate potential confounding influences.

### 3.3. Measures

Manipulation Checks This study used six items for assessing personification, four on the positive and negative frame and three for assessing cartoon children voices. To examine anthropomorphism manipulation, six items were selected from Newton et al. [81], e.g., “The alarm clock/moon appears to have emotions and feelings”, using a 7-point Likert scale (1 = strongly disagree; 7 = strongly agree). Four items were selected from the study by Cho et al. [55] to examine the operation of positive and negative frames. For the checking of positive frames, two items are used. For example, “This cartoon focuses on the benefits of healthy sleep”. For the negative frame check, two items were used. For example, “This cartoon focuses on the downsides of sleep deprivation”. Items were rated on seven-point Likert scales of 1 (strongly disagree) to 7 (strongly agree). Regarding the rating of the cartoon children voices, three items, including “The voice tones of this cartoon are cartoon”, were scored using a 7-point Likert scale.

Cartoon Attitude We selected five items from Sundar and Kim [82] and adapted them appropriately, such as “How interesting do you think this cartoon is?” and “Do you think this cartoon is convincing?” These were rated using a 7-point Likert scale, and the scale maintained excellent internal consistency (α = 0.73).

Risk Perception The five items of the Risk Perception Scale were taken from studies by Witte et al. [83] and Guan and So [40] with slight modifications. A seven-point Likert scale was used to assess viewers’ perceptions of the risk of unhealthy sleep, such as “If I don’t pay attention to my sleep, I may experience health problems” and “When sleep is insufficient, there may be serious health problems”. Averaging these five items yielded a risk perception index for unhealthy sleep that was in good agreement (α = 0.77).

Behavioral Intentions To assess healthy sleep intentions, two items were selected from previous related research [84]. Viewers were asked to answer on a seven-point Likert scale (1 = strongly disagree; 7 = strongly agree) the items “I plan to sleep 8–9 h most nights of the week” and “I will sleep most nights of the week 8–9 h”. The Healthy Sleep Behavior Intention Scale also reported excellent agreement (α = 0.82).

### 3.4. Procedure

**Figure 1:**
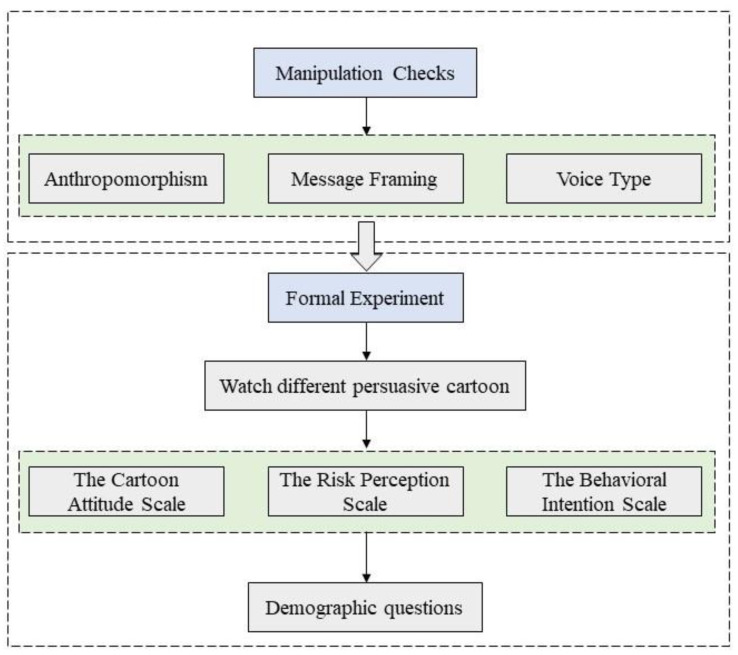
The research procedure framework.

Before conducting the formal experiment, we recruited 75 participants for independent variable examination using the seven-point Likert scale to assess whether the various levels of the independent variables were entirely different (Figure 1). The independent-samples *t*-test revealed significant differences between the levels of the variables, and the data results showed that the control of the independent variables was successful and that the independent variables at each level were fully distinguished.

In the formal experiment, we invited 222 subjects to participate by issuing questionnaires. After reading the participants’ informed consent, they were randomly assigned to one of eight groups. First, participants were asked to watch the persuasive cartoon of the corresponding group. Second, they were asked to answer (1) the Cartoon Attitude Scale, (2) the Risk Perception Scale, (3) the Behavioral Intention Scale, and three sets of questions. Finally, participants answered demographic questions (e.g., age, gender, and education).

## 4. Result

### 4.1. The Sample Profile

A total of 222 questionnaires were distributed in this experiment. After excluding 15 invalid questionnaires, 207 valid questionnaires were included in the final data set for analysis. Descriptive statistics showed that 56.5% of the participants were between the ages of 23 and 26, 49.3% were female, and 50.7% were male. Regarding academic qualifications, 55.6% of the participants have a bachelor’s degree, and 39.2% are postgraduates. The specific results are shown in Table 2.

### 4.2. Manipulation Checks

Participants who watched anthropomorphic cartoons rated their perception of anthropomorphism (M = 5.35, SD = 0.55) higher (t [73] = 14.46, *p* < 0.001) than those who watched non-anthropomorphic cartoons (M = 3.40, SD = 0.58). Participants viewing the positive-frame cartoons reported more attention to the positive outcomes of healthy sleep (M_positive_ = 5.30, SD = 0.57, M_negative_ = 3.39, SD = 0.40, t [30] = 11.82, *p* < 0.001), while participants viewing the negative-frame cartoons reported more focus on unhealthy sleep results (M_negative_ = 5.56, SD = 0.77 vs. M_positive_ = 2.51, SD = 0.64, t [41] = 12.82, *p* < 0.001). In addition, participants who listened to the cartoon children’s voice had a higher perception of a cartoon voice (M = 5.91, SD = 0.45) (t [73] = 21.12, *p* < 0.001) than those who listened to the adult female’s speech (M = 3.48, SD = 0.53). Therefore, the personification, framing, and voice manipulation were successful.

### 4.3. Hypothesis Testing 

A three-way analysis of variance (ANOVA) was performed with anthropomorphism, message framing, and voice type as independent variables and cartoon attitude, risk perception, and behavioral intention as dependent variables. The results showed that the main effect of anthropomorphism on cartoon attitude was significant (F(1,199) = 4.72, *p* < 0.05). There was a significant two-way interaction effect between anthropomorphism and frame on cartoon attitude (F(1,199) = 11.41, *p* = 0.001). There was also a significant three-way interaction effect between frame, anthropomorphism, and voice type on risk perception (F(1,199) = 5.89, *p* < 0.05). The results are shown in Table 3.

Consistent with H1 and H2, participants who watched anthropomorphic cartoons (M = 4.55, SD = 1.04) showed higher cartoon attitudes (F(1,199) = 4.72, *p* < 0.05), whereas anthropomorphism had no main effect on behavioral intention (F(1,199) = 0.02, *p* = 0.89). Message framing was not significantly different for cartoon attitudes (F(1,199) = 1.17, *p* = 0.28) and behavioral intentions (F(1,199) = 0.59, *p* = 0.81).

**Figure 2:**
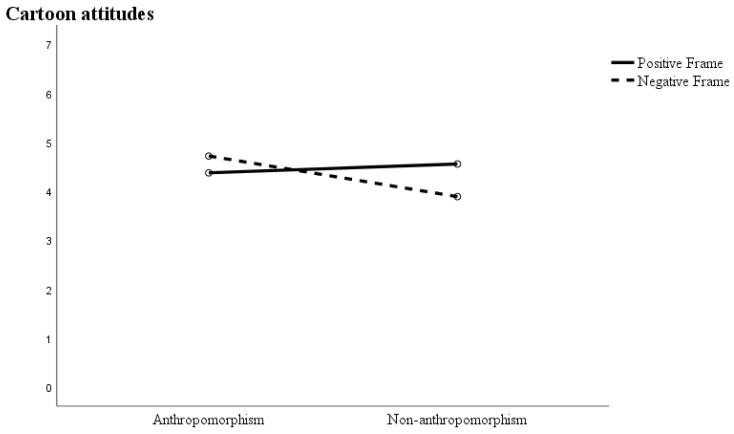
The effect of message frame on cartoon attitudes by anthropomorphism.

The experimental results show that H3 is correct. Message framing and anthropomorphism had a significant interaction with viewers’ attitudes toward cartoons (F(1,199) = 14.78, *p* < 0.00) but no significant interaction with behavioral intentions (F(1,199) = 0.14, *p* = 0.71). Specifically, for viewers viewing negative frame information, the anthropomorphic cartoon (M = 4.72, SD = 0.96) had higher cartoon attitude scores than the non-anthropomorphic (M = 3.89, SD = 0.94) cartoon. The two-way interaction results of anthropomorphism and framing effect on cartoon attitude are shown in Figure 2.

**Figure 3:**
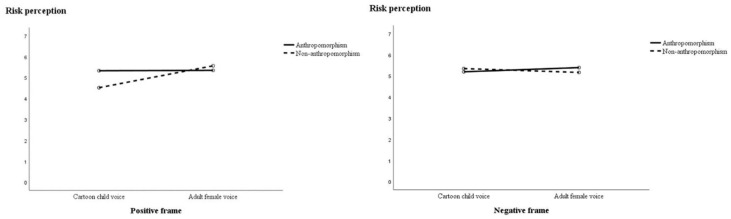
The effects of anthropomorphism on risk perception by voice type and message frame.

For H4, the results showed no significant interaction effects between framing, anthropomorphism and voice type on cartoon attitudes (F(1,199) = 0.35, *p* = 0.56) or behavioral intention (F(1,199) = 3.187, *p* = 0.08), and there was a significant three-way interaction of risk perception (F(1,199) = 5.89, *p* < 0.05), partially supporting H4. Further research showed that participants viewing positive frames reported higher scores for the anthropomorphic (M = 5.33, SD = 1.01) cartoon than the non-anthropomorphic cartoon (M = 4.52, SD = 1.05; F(1,199) = 8.75, *p* < 0.01). However, when the participants were exposed to adult female voices, the anthropomorphic cartoon (M = 5.35, SD = 1.05) scored no better than the non-anthropomorphic cartoon (M = 5.57, SD = 0.94; F(1,199) = 0.56, *p* = 0.46) with a strong risk perception. Figure 3 illustrates the results.

### 4.4. Conditional Process Analysis

**Figure 4:**
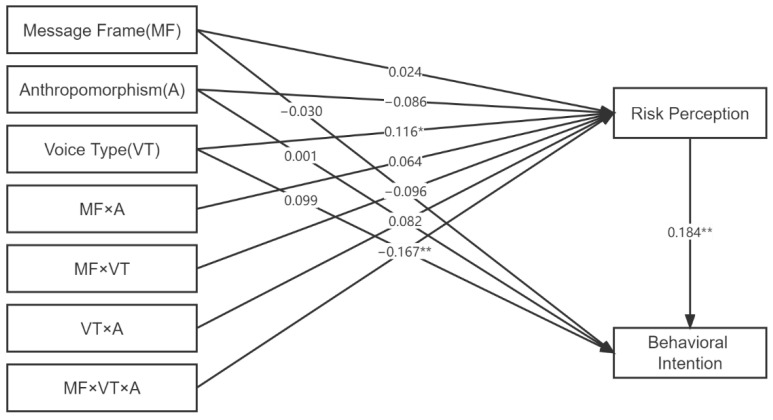
The research framework and path coefficients (the standardized regression coefficient). Note: * *p* ≤ 0.10; ** *p* ≤ 0.05.

To test the mediation moderation model, we used PROCESS SPSS macro [80] with a sample size of 5000 and a confidence interval of 95%. The models in this study included message framing (positive frame = 0; negative frame = 1), anthropomorphism (anthropomorphic = 0, non-anthropomorphic = 1), and voice type (cartoon child voice = 0, adult female voice = 1). We also tested interactions between two and three variables. The results showed that: (a) the interaction effect of message frame × voice type × anthropomorphism was significant (β = −0.167, *p* = 0.016, LLCI = −0.303 to ULCI = −0.031); (b) risk perception was mediated by the interaction effects of frame × voice type × anthropomorphism and behavioral intention (β = −0.031, BootLLCI = −0.075 to BootULCI = −0.002), which is consistent with H5, and the specific results are shown in Figure 4. Furthermore, we found that H2 is also supported in the mediation model: (a) Behavioral intentions increased with risk perception (β = 0.184, *p* = 0.008, LLCI = 0.048 to ULCI = 0.320), (b) while non-anthropomorphic images with a cartoon child’s voice weakened the effect of message framing on risk perception (β = −0.167, *p* = 0.016, BootLLCI = 0.007 to BootULCI = 0.179). Therefore, message framing has no main effect on behavioral intention, which supports H2.

## 5. Discussion

This study examines the effects of anthropomorphic interactive persuasive interfaces on healthy sleep among young users. This study explores how anthropomorphism, message framing, and voice type are combined. The results show that: (1) Anthropomorphism affected attitude about the cartoon but did not affect behavioral intention. (2) Message framing did not affect attitude or behavior intention. (3) Message framing and anthropomorphism had an interactive effect on attitude but not on behavioral intention. (4) Anthropomorphism, message framing, and voice type had a triple interaction effect on risk perception, and risk perception mediated the interaction effect of anthropomorphism, message framing, and voice type on behavioral intention.

This study found that compared with non-anthropomorphic design, the interactive persuasion interface with anthropomorphic design caused users to have more positive attitudes and attracted more users [85,86]. This suggests that the alarm clock and the moon that add human-like features such as eyes and expressions to the interactive persuasion interface may endow the viewer with sensory associations [34], in turn activating the inanimate object’s life-oriented intention association [36]. Regarding the main effect of anthropomorphism on attitudes, we reach the same conclusion as Burgoon et al. [52] in the anthropomorphic task interface. However, we did not find a significant effect of anthropomorphism on behavioral intentions [87]: Neither anthropomorphic nor non-anthropomorphic interactive persuasion interfaces could produce behavioral intentions for healthy sleep. This may be because anthropomorphic design has an immediate characteristic for sensory stimuli, so it is not difficult to explain the main effect of anthropomorphism on cartoon attitude. However, behavioral change is multifactorial (e.g., encompassing assessments of outcomes, motivations, and other perceived facilitators), Therefore, we could not find in our brief experiments a main effect of anthropomorphism on behavioral attitudes. However, in social psychology research, attitudes and behavioral intentions have the characteristics of consistency and convergence. Therefore, we can guess that based on a user’s positive attitude toward an interactive cartoon, repeated stimulation (e.g., prolonged exposure to anthropomorphic interactive cartoon) may lead to the establishment of the user’s healthy sleep intention and behavioral changes and ultimately achieve the influence of anthropomorphism on to behavioral intentions.

Message framing had no main effect on attitudes or behavioral intentions. There were no significant differences in attitudes or behavioral intentions whether viewers were exposed to positive framing information (the benefits of healthy sleep) or negative framing information (the disadvantages of unhealthy sleep). This finding is unexpected but not surprising, as similar results were found in a past study by Arora et al. [64]. In addition, de Bruijn et al. [63] also did not find a main effect of message framing on behavioral intentions for health interventions, although they found that a combination of message framing and temporal distance influences behavioral intentions in adults. This suggests that it is difficult for message framing alone to play a major role but that when it is combined with other factors (such as temporal distance), message framing may play a role in influencing behavioral intentions. That said, message framing is a design method that im-plies content and is weak in perception; it needs to be presented in good synergy with visual and auditory signals.

Although few studies have mentioned the interaction of anthropomorphism and message framing, we found that anthropomorphism and message framing significantly interact with attitudes in our study. Further, for viewers exposed to negative framing information, using an anthropomorphic interface resulted in higher attitude scores. This may be because under the negative frame, the anthropomorphic consequences (red eyes, dizziness) are more clearly articulated for unhealthy sleep than the non-anthropomorphic ones (smoke alarm clock) [88], and the presentation is more visually impactful and persuasive [47], resulting in a more positive attitude from viewers. In addition, we did not find an interaction effect between anthropomorphism and message framing on behavioral intentions. This shows that although the expressiveness and visual impact of the message framing can be enhanced through anthropomorphism, the effect of anthropomorphism alone may not improve users’ behavioral intention for healthy sleep.

We found that anthropomorphism, message framing, and voice type significantly influenced risk perception. In particular when the participants watched the interactive cartoon with positive framing information, those who received cartoon voices were more aware of the risk of anthropomorphic transmission. There may be several reasons for this effect. First, anthropomorphism establishes an emotional connection between viewers and interactive cartoons [65], which leads to greater interest in viewing information. Second, the matching and consistency of anthropomorphic interface design and cartoon children’s speech enhance the viewer’s sense of substitution and feeling [71]. Finally, the finding might have been because positive framed information enhances users’ risk perception of unhealthy sleep through guilt [54]. In addition, this study verified the mediating effect of risk perception on behavioral intentions. Consistent with previous findings by Gursoy et al. [58] and Jin and Atkinson [79], risk perception predicted behavioral intentions, that is, risk perception mediates the effect of anthropomorphism on behavioral intention. There was a three-fold interaction effect of anthropomorphism, message framing, and voice type on behavioral intentions. In other words, when people felt the downsides of unhealthy sleep, their willingness to perform healthy behaviors increased.

### 5.1. Theoretical Implications

In recent years, anthropomorphism research has received considerable attention, focusing mainly on consumer attitudes toward anthropomorphic advertising, artificial intelligence, and product purchase and use intentions. However, current research on anthropomorphism’s role in public health communication is still in its infancy and has just attracted the attention of scholars. This research is based on a previous information design of the two factors of message framing and voice type. It innovatively integrates the design element of anthropomorphism, forming a new effective information combination method. We found that the effective combination of the three factors can induce risk perception to guide potential behavioral intentions and innovatively provides theoretical and practical support.

### 5.2. Practical Implications

This study found that positive descriptions of the benefits of healthy sleep elicited higher risk perceptions and promote healthier behavioral intentions than negative descriptions of the disadvantages of unhealthy sleep, especially when paired with message-framed text with anthropomorphic graphic designs and voice backgrounds. Therefore, in designing unhealthy sleep risk information, interaction designers should consider using anthropomorphic techniques to convey the benefits of healthy sleep to users with cartoon child-type background sounds. However, in terms of enhancing the attractiveness of the interactive interface to users, anthropomorphic graphic design, especially the combination of anthropomorphic graphics and negative information text, can attract users’ attention and enhance users’ positive attitudes toward the interactive interface.

### 5.3. Limitations and Directions for Future Research

Although this research has theoretical and practical significance, it is not without limitations.

(1) From the experimental setting point of view, due to the setting and our possibly limited resources to develop the material, we were unable to measure how effective the interactive animation was compared with the blank control. In future research, (1) we will incorporate quantitative measures of the control group and before-and-after comparisons to better compensate for our deficiencies; (2) we may have a phased tracking of behaviors to enrich the quantitative measures of before-and-after comparisons.

(2) From a design point of view, this experimental design inserted interactive cartoons into the questionnaire. However, in the actual application scenario, the entertainment habits of young users before going to bed are based on electronic products such as mobile phones and tablets. Therefore, future research should consider how to better integrate interactive cartoons with specific products, applications, and usage scenarios in design.

(3) From the participants’ aspect, to ensure that the experimental materials and the quality of the questionnaire data would be fully understood, our questionnaires was mainly distributed to users with a bachelor’s degree or above. Thus, we could only generalize the findings to the population of youth with a bachelor’s degree.

(4) From the experimental procedure aspect, we lacked the preliminary investigation of the baseline and cycle of the participants’ sleep cycles, and we failed to quantify the improvements in behavioral intentions. Baseline measurements will be taken in future base case studies.

(5) From the user’s point of view, the scope of the youth group is enormous. In addition, there are various reasons for lack of sleep among young people related not only to their behavior habits but also to the influence of the external environment. Therefore, we cannot determine the generalizability of the findings of this experiment to youth groups with different attributes (gender, region, etc.). The future design of a healthy sleep persuasion interface should be more detailed and targeted to carry out more in-depth research on certain types of youth groups. Finally, we only focus on the present impacts on users’ healthy sleep intentions. How future anthropomorphic design can guide users in conducting behavioral interventions and follow-up monitoring should be further explored.

## 6. Conclusions

This study is the first to investigate anthropomorphism, message framing, and voice type in the field of health persuasion, aiming to guide the design of effective sleep persuasion interfaces. Specifically, we found that increasing the risk perception of unhealthy sleep can effectively improve users’ healthy sleep behavior intentions. Anthropomorphism, message framing, and voice type have a triple interaction effect on risk perception. Anthropomorphism and message framing have positive impacts on user attitudes. This research may contribute to our recreational product reminder program, so more attention should be paid to anthropomorphic usage scenarios during the design phase. For example, when using anthropomorphic design to improve a user’s healthy sleep behavior intention, it is best to match the positive frame text and cartoon children voices. To capture the user’s attention, a combination of anthropomorphic graphic design and negative frame descriptions should be used.

## Figures and Tables

**Table 1:** The experimental stimulus designs.

Independent Variable	Level	
Anthropomorphic	**Non-anthropomorphic**	**Anthropomorphic**
	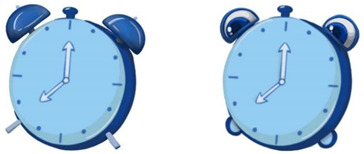	
	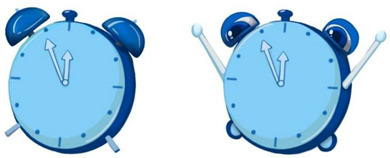	
	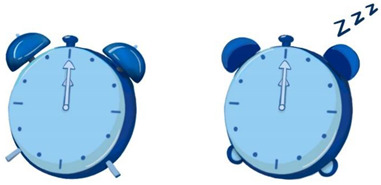	
	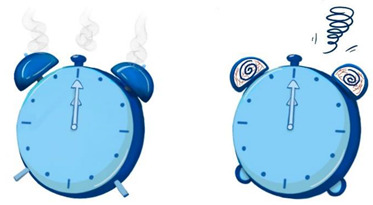	
Message framing	Positive frame	Negative frame
	Good sleep is good for physical and mental health.	Lack of sleep is harmful to physical and mental health.
Voice type	Cartoon child voice	Adult female voice
	Healthy sleep, developing a scientifically regular life, and rest are beneficial to physical and mental health	Lack of sleep, inability to develop a scientifically regular life, and lack of rest will harm physical and mental health

**Table 2:** The sample profile.

Characteristics	N	Percentage
Gender		
Male	105	50.7
Female	102	49.3
Age		
15–18 years	1	0.5
19–22 years	85	41.1
23–26 years	117	56.5
28–30 years	2	1.0
31–34 years	2	1.0
Education		
High school/Secondary school/Technical school student	2	1.0
College students	9	4.3
Undergraduate student	115	55.6
Graduate student	79	38.2
Doctoral student	2	1.0

**Table 3:** Effects on cartoon attitudes, risk perception, and behavioral intention.

Sources of Variation		Cartoon Attitudes		Risk Perception		Behavioral Intention	
		F Value	η^2^	F Value	η^2^	F Value	η^2^
Anthropomorphism (A)	(1,199)	4.72 **	0.02	1.32	0.01	0.02	0.00
Message Frame (MF)	(1,199)	1.17	0.01	0.34	0.00	0.06	0.00
Voice Type (VT)	(1,199)	1.16	0.01	3.47 *	0.02	3.38 *	0.02
A × MF	(1,199)	11.41 ****	0.05	0.79	0.00	0.14	0.00
A × VT	(1,199)	0.12	0.00	1.25	0.01	1.30	0.01
MF × VT	(1,199)	0.08	0.00	3.25 *	0.02	3.02 *	0.02
A × MF × VT	(1,199)	0.35	0.00	5.89 **	0.03	3.19 *	0.02

* p ≤ 0.10; ** *p* ≤ 0.05; **** *p* ≤ 0.001.

**Figure A1:**
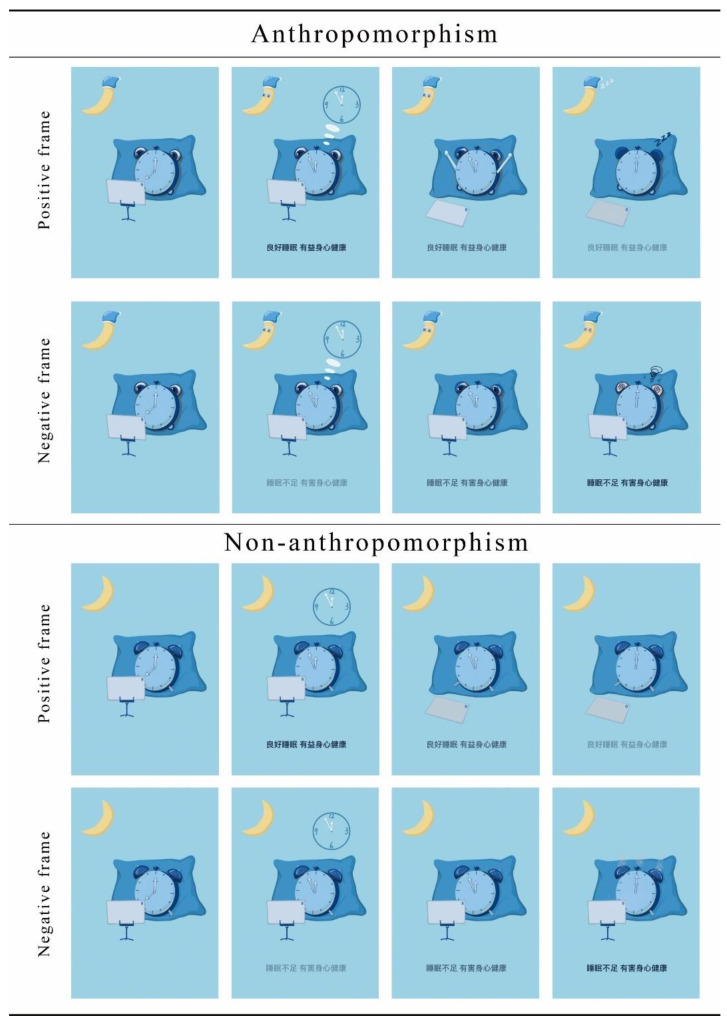
The experimental material. 睡眠不足 有害身心健康: Lack of sleep is harmful to physical and mental health. 良好睡眠 有益身心健康: Good sleep is good for physical and mental health.

## Data Availability

The data presented in this study are available on request from the corresponding author. The data are not publicly available due to restrictions.

## Supplementary Materials

The following supporting information can be downloaded at: https://www.mdpi.com/article/10.3390/ijerph19159570/s1.
